# An effective solution for analyzing the electromagnetic scattering characteristics of composite conducting dielectric objects under multiple angle incidence

**DOI:** 10.1038/s41598-025-11786-1

**Published:** 2025-07-23

**Authors:** Meng Kong, Xin Yuan Cao, Qi Qi, Xian Liang Wu

**Affiliations:** Anhui Province Key Laboratory of Simulation Calculation and Design for Electronic Information System, Hefei Normal University, Hefei, 230601 China

**Keywords:** Composite conducting-dielectric objects, Electromagnetic scattering, Compressive sensing, Electrical and electronic engineering, Computational science

## Abstract

The accurate modeling of electromagnetic scattering from composite conducting-dielectric objects under multi-angle incidence is crucial in the field of electromagnetic engineering, however, due to the huge computational resources required, there is a disadvantage of slow analysis speed on personal computer platforms. Based on the compressive sensing theory, this article constructs a new excitation source solution model using the Volume-Surface Integral Equation platform. The special CS technique is separately applied to the conductive surface and dielectric region of the composite object, respectively, in order to improve the efficiency of analyzing the wide-angle electromagnetic scattering characteristics of the composite object. Compared with the traditional Volume-Surface Integral Equation method, the proposed method can significantly reduce the number of repeated calculations of matrix equations while maintaining the same level of accuracy and memory consumption, so as to accelerate the overall calculation of the wide-angle electromagnetic scattering. By the complexity analysis of the proposed algorithm and the numerical simulation of various composite objects with different materials and structures, the efficiency of the proposed algorithm is verified.

Recently, the analysis of electromagnetic (EM) scattering characteristics from composite conducting-dielectric objects has generated significant research interest due to its extensive applications in biological engineering^1,2^ target detection^3^ and Metasurface device design^4,5^. To accurately evaluate the EM response signal of composite objects from multiple dimensions, it has become important to model the EM scattering at various angles of incidence. However, the efficient calculation of its multi-angle scanning response has always been a difficult problem in the field of electromagnetics. The challenge comes from two main aspects, one is the accurate electromagnetic modeling of heterogeneous scatterers, and the other is the fast calculation of multi-angle incidence to the targets.

For the first problem, solving surface integral equation (SIE)^6,7^ or volume-surface integral equation (VSIE)^8,9^ by moment of method (MoM) is typical computational model. Compared with SIE method, VSIE method is more convenient for solving inhomogeneous composite objects with arbitrary shape. This is thanks to VSIE method applies volume integral equation (VIE) and SIE to dielectric region and conducting surface respectively, and does not need special treatment for junctions of different materials, which has the advantages of simple implementation. However, the large unknown quantity brought by the volume division of the medium region leads to the high cost of computing resources, which shows the weakness of traditional VSIE method, especially for electrical large-scale structures.

For fast computational problem of wide-angle electromagnetic scattering from Composite Conducting-Dielectric Objects, such as multilevel fast multipole method (MLFMM)^10,11^ the precorrected-FFT (P-FFT) method^12^ adaptive integral method (AIM)^13^, the nested equivalent source approximation (NESA) method^14^ can be used for traditional VSIE method to accelerate the solution of the matrix equation under the excitation of each interesting incident angle, however, the above fast algorithms have to solve the matrix equation one by one according to the incidence angles for obtaining the responses over a wide sweep angle. In order to reduce the number of repeated calculations for the wide-angle scattering problem, Compressed Sensing (CS) theory has been applied to MoM^15^ for improving the efficiency of wide-angle electromagnetic scattering analysis of conductor targets^16,17^ and inhomogeneous dielectric objects^18^ respectively. Unfortunately, for wide-angle EM scattering of the composite conducting-dielectric objects, especially for solving VSIE by MoM, a special excitation source solution model has not yet been constructed for improving the computational efficiency.

In the proposed calculation method of this paper, a new excitation source based on observing all incident angle information is implanted into the matrix equation obtained by VSIE-MoM, and the measurement value can be obtained after only a fewer calculations of above equation, which is much less than the number of the repeated calculation for each incident Angle in the traditional VSIE method. Then, according to the structure characteristics of the composite conducting-dielectric objects, the conductive surface and dielectric region from the composite objects are treated respectively in the aspect of CS technology to ensure the high sparsity of the unknowns. Finally, all the responses to be reconstructed accurately with the help of the recovery algorithm.

## Solving the Volume-Surface integral equation by MoM

Consider a composite conducting-dielectric object illuminated by an incident plane wave ***E***^i^, in which the dielectric region is composed of non-magnetic materials (*µ* = *µ*_0_). According to the equivalence principle, the conducting bodies and the dielectric materials are replaced by surface current ***J***_S_ and volume current ***J***_*V*_ in free space, respectively.

The total electric field ***E***(**r**) is the sum of the incident field ***E***^i^ (**r**) and scattered fields ***E***^S^ (**r**), namely,

1 $$E\left( {\mathbf{r}} \right)\,=\,{E^{\text{i}}}\left( {\mathbf{r}} \right)\,+\,{E^{\text{S}}}\left( {\mathbf{r}} \right)$$

For conducting surface *S*, tangential electric field is zero enforced by boundary conditions, i.e.,

2a $${[{E^{\text{i}}}\left( {\mathbf{r}} \right)\,+\,{E^{\text{S}}}\left( {\mathbf{r}} \right)]_{{\text{tan}}}}=0;{\mathbf{r}} \in S$$

In the dielectric regions *V*, considering the constitutive equation ***D***(***r***)=ε(**r**)***E***(**r**), Eq. (1) can be rewritten as

2b $$[{E^{\text{i}}}\left( {\mathbf{r}} \right)\,+\,{E^{\text{S}}}\left( {\mathbf{r}} \right)]\,=\,{D_V}\left( {\mathbf{r}} \right)/{\text{e}}\left( {\mathbf{r}} \right);{\mathbf{r}} \in V$$

where ε(**r**) denote the permittivity of the dielectric volumes, and ***D***_*V*_(**r**) is electric flux density, whose relation to ***J***_*V*_ can be expressed as

3 $${J_V}\left( {\mathbf{r}} \right)\,=\,j \omega {\text{k}}\left( {\text{r}} \right){{\mathbf{D}}_V}\left( {\mathbf{r}} \right)$$

in which κ(r)=(ε(**r**)- ε_0_)/ε(**r**), ε(**r**)=ε_0_ε_r_(**r**).

As we known, the scattered field ***E***^S^ (**r**) is generated by ***J***_S_ and ***J***_*V*_, which can be shown as

4 $${{\varvec{E}}^S}\left( {\mathbf{r}} \right)= - j\omega {{\varvec{A}}_S}\left( {\mathbf{r}} \right) - \nabla {\Phi _S}({\mathbf{r}}) - j\omega {{\varvec{A}}_V}\left( {\mathbf{r}} \right) - \nabla {\Phi _V}({\mathbf{r}})$$

in which ***A***_*S*_(**r**), ***A***_*V*_(**r**), *Φ*_*S*_(**r**), and *Φ*_*V*_(**r**) are the vector magnetic potential and scalar potential excited by ***J***_*S*_ and ***J***_*V*_, respectively, which can be formulated by

5 $$\begin{gathered} {{\varvec{A}}_u}\left( {\mathbf{r}} \right)=\frac{{{\mu _0}}}{{4\pi }}\int_{u} {{{\varvec{J}}_u}({\mathbf{r^{\prime}}})} Gdu^{\prime}\begin{array}{*{20}{c}} {}&{u=S,V} \end{array} \hfill \\ {\Phi _u}({\mathbf{r}})= - \frac{1}{{4\pi j\omega {\varepsilon _0}}}\int_{u} {\nabla \cdot {{\varvec{J}}_u}({\mathbf{r^{\prime}}})} Gdu^{\prime}\begin{array}{*{20}{c}} {}&{u=S,V} \end{array} \hfill \\ \end{gathered}$$

where $G=\frac{{{e^{ - j{k_0}|{\mathbf{r}} - {\mathbf{r^{\prime}}}|}}}}{{|{\mathbf{r}} - {\mathbf{r^{\prime}}}|}}$ denote the scalar Green function in free space.

By substituting Eqs. (3)–(5) into Eq. (2), the electric field VSIE is constructed in terms of ***J***_*S*_ on the conductive surface and ***D***_*V*_ in the dielectric region to characterize the electromagnetic scattering from the composite conductive dielectric object.

For solving VSIE, under the operating framework of MoM, the conductive surface *S* and the dielectric region *V* are divided by triangular patches and tetrahedral elements respectively, and ***J***_*S*_ and ***D***_*V*_ are discretized by RWG basis functions *f*^*S*^ and SWG basis functions *f*^*V*^ respectively.

6 $$\begin{gathered} {{\varvec{J}}_S}\left( {\mathbf{r}} \right)=\sum\limits_{{m^{\prime}=1}}^{{{N_S}}} {I_{{m^{\prime}}}^{S}} f_{{m^{\prime}}}^{s}({\mathbf{r}}) \hfill \\ j\omega {{\varvec{D}}_V}({\mathbf{r}})=\sum\limits_{{m^{\prime}=1}}^{{{N_V}}} {I_{{m^{\prime}}}^{V}} f_{{m^{\prime}}}^{V}({\mathbf{r}}) \hfill \\ \end{gathered}$$

in which, *N*_*S*_ is the number of edges from triangular patches that make up the *S*, and *N*_*V*_ is the number of faces from tetrahedral elements that make up the *V.*

With the help of the Galerkin testing, the VSIE can be rewritten in matrix form as

7 $$\left[ {\begin{array}{*{20}{c}} {{\varvec{Z}}_{{{N_S} \times {N_S}}}^{{SS}}}&{{\varvec{Z}}_{{{N_S} \times {N_V}}}^{{SV}}} \\ {{\varvec{Z}}_{{{N_V} \times {N_S}}}^{{VS}}}&{{\varvec{Z}}_{{{N_V} \times {N_V}}}^{{VV}}} \end{array}} \right]\left[ {\begin{array}{*{20}{c}} {{\varvec{I}}_{{{N_S} \times 1}}^{S}} \\ {{\varvec{I}}_{{{N_V} \times 1}}^{V}} \end{array}} \right]=\left[ {\begin{array}{*{20}{c}} {{\varvec{V}}_{{{N_S} \times 1}}^{S}} \\ {{\varvec{V}}_{{{N_V} \times 1}}^{V}} \end{array}} \right]$$

where ${{\varvec{V}}^t}(t=S,V)$ and $\:{{\varvec{I}}^u}(u=S,V)\:$ are the known excitation and the unknown expansion coefficient, respectively. ${{\varvec{Z}}^{tu}}(t,u=S,V)\:$ is the subdomain impedance matrix denoting the excitation from *t* part which has impact on *u* part.

Considering the electromagnetic scattering problem under multi-angle incident, Eq. (2) can be shown as

8 $$\left[ {\begin{array}{*{20}{c}} {{\varvec{Z}}_{{{N_S} \times {N_S}}}^{{SS}}}&{{\varvec{Z}}_{{{N_S} \times {N_V}}}^{{SV}}} \\ {{\varvec{Z}}_{{{N_V} \times {N_S}}}^{{VS}}}&{{\varvec{Z}}_{{{N_V} \times {N_V}}}^{{VV}}} \end{array}} \right]\left[ {\begin{array}{*{20}{c}} {{\varvec{I}}( \theta ) _{{{N_S} \times n}}^{S}} \\ {{\varvec{I}} (\theta)_{{{N_V} \times n}}^{V}} \end{array}} \right]=\left[ {\begin{array}{*{20}{c}} {{\varvec{V}} (\theta)_{{{N_S} \times n}}^{S}} \\ {{\varvec{V}} ( \theta)_{{{N_V} \times n}}^{V}} \end{array}} \right]$$

in which *θ* denote incident angle, *n* is the number of incidence angles, and it is worth noting that ${{\varvec{Z}}^{tu}}(t,u=S,V)$ is an impedance matrix independent of *θ*.

For the solution of Eq. (8), the traditional method needs to repeat the calculation of Eq. (7) for *n* times under the irradiation of each incident angle. Even if the single solution of the matrix Eq. (7) is accelerated by the fast algorithm mentioned in the first paragraph, it still consumes a huge amount of computational resources when the scanning angle of incident is very wide.

## Improving computational efficiency by CS

To reduce the number of repeated calculations of the electromagnetic scattering matrix equation from Composite Conducting-Dielectric Objects over wide incident angle, an efficient analysis method based on compressed sensing is constructed, and the specific implementation process is as follows.

### [Step1] constructing the excitation source including all incidence angle information

The multiple excitation term on the right-hand side of Eq. (8) can be modified as

9 $${\varvec{V}}^{\prime}{(\theta )_{({N_S}+{N_V}) \times m}}={\varvec{V}}{(\theta )_{({N_S}+{N_V}) \times n}}{\varvec{\Phi}_{n \times m}}\begin{array}{*{20}{c}} {}&{(m<n)} \end{array}$$

in which ${{\mathbf{\Phi }}_{n \times m}}$ is considered to be a measurement matrix, $${\varvec{V}}(\theta )=\left[ {\begin{array}{*{20}{c}} {{\varvec{V}}(\theta )_{{{N_S} \times n}}^{S}} \\ {{\varvec{V}}(\theta )_{{{N_V} \times n}}^{V}} \end{array}} \right]$$ is actual multi-angle excitation term. $\:{\varvec{V}}^{\prime}{(\theta )_{({N_S}+{N_V}) \times m}}$ denote the combination of the column vector $${\varvec{V}}_{i}^{\prime }{(\theta )_{({N_S}+{N_V}) \times 1}}\begin{array}{*{20}{c}} {}&{(i=1,2,3,4,...,m)} \end{array}$$ including all incident angle information.

### [Step 2] rewriting multi-angle matrix equations in the CS framework

Equation (9) is implanted in the Eq. (8), and the equation can be given as

10 $${{\varvec{Z}}_{({N_S}+{N_V}) \times ({N_S}+{N_V})}}{\varvec{I}}^{\prime}{(\theta )_{({N_S}+{N_V}) \times m}}={\varvec{V}}^{\prime}{(\theta )_{({N_S}+{N_V}) \times m}}$$

Considering the linear property of Eq. (10), the response ${\varvec{I}}^{\prime}{(\theta )_{({N_S}+{N_V}) \times m}}\:$ can also be expressed in a form similar to Eq. (9) as

11 $${\varvec{I}}^{\prime}{\left( {\mathbf{\theta }} \right)_{\left( {{N_S}+{N_V}} \right) \times m}}={\varvec{I}}{\left( {\mathbf{\theta }} \right)_{\left( {{N_S}+{N_V}} \right) \times n}}{{\mathbf{\Phi }}_{n \times m}}=\varvec{\alpha}{\left( {\mathbf{\theta }} \right)_{\left( {{N_S}+{N_V}} \right) \times n}}{({\varvec{\varPsi}_{n \times n}})^{\varvec{T}}}{{\mathbf{\Phi }}_{n \times m}}$$

where the actual response ${\varvec{I}}(\theta )$ can be shown as

12 $${\varvec{I}}(\theta )=\left[ {\begin{array}{*{20}{c}} {{\varvec{I}}(\theta )_{{{N_S} \times n}}^{S}} \\ {{\varvec{I}}(\theta )_{{{N_V} \times n}}^{V}} \end{array}} \right]=\varvec{\alpha}{\left( {\mathbf{\theta }} \right)_{\left( {{N_S}+{N_V}} \right) \times n}}{({\varvec{\varPsi}_{n \times n}})^{\varvec{T}}}$$

in which the unknown $\varvec{\alpha}{\left( {\mathbf{\theta }} \right)_{\left( {{N_S}+{N_V}} \right) \times n}}$ is the sparse representation of $\:\:{\varvec{I}}{(\theta )_{_{{\left( {{N_S}+{N_V}} \right) \times n}}}}$ in ${\varvec{\varPsi}_{n \times n}}$.

Inspired by the CS framework, in Eq. (11), ${{\mathbf{\Phi }}_{n \times m}}$ and ${\varvec{\varPsi}_{n \times n}}$ are considered to be measurement matrix and sparse transformation matrix respectively. Based on the CS theory, the measurement matrix ${{\mathbf{\Phi }}_{n \times m}}$ must satisfy the Restricted Isometry Property (RIP) property^19^ that is, the incoherence between ${{\mathbf{\Phi }}_{n \times m}}$ and ${\varvec{\varPsi}_{n \times n}}$ must be guaranteed, so that the unknown sparse signal $\varvec{\alpha}{\left( {\mathbf{\theta }} \right)_{\left( {{N_S}+{N_V}} \right) \times n}}$ can be accurately reconstructed. Since its randomness, the Gaussian random matrix composed of random distribution elements can ensure its incoherence with ${\varvec{\varPsi}_{n \times n}}$ matrix, so Gaussian random matrix can be qualified for the role of measurement matrix ${{\mathbf{\Phi }}_{n \times m}}$.Generally, the sparse transformation matrix ${\varvec{\varPsi}_{n \times n}}$ composed of complete orthogonal basis can realize the sparsity of ${\varvec{I}}(\theta )$^20^ and the selection of the specific ${\varvec{\varPsi}_{n \times n}}$ needs to be determined according to the angular response characteristics predicted by the construction of analyzed targets. ${\varvec{I}}^{\prime}{\left( {\mathbf{\theta }} \right)_{\left( {{N_S}+{N_V}} \right) \times m}}$ is the measurement value of sparse vector $\varvec{\alpha}{\left( {\mathbf{\theta }} \right)_{\left( {{N_S}+{N_V}} \right) \times n}}$. It is worth noting that ${\varvec{I}}^{\prime}{\left( {\mathbf{\theta }} \right)_{\left( {{N_S}+{N_V}} \right) \times m}}$ is the response including all incident angle information under the excitation of ${\varvec{V}}^{\prime}{(\theta )_{({N_S}+{N_V}) \times m}}$, and one can be obtained by solving Eq. (10), which requires repeating the precorrected FFT method *m* times (*m* < *n*).

To independently describe the conductor surface measurement ${\varvec{I}}^{\prime}(\theta )_{{_{{{N_S} \times m}}}}^{S}$ and the dielectric region measurement ${\varvec{I}}^{\prime}(\theta )_{{_{{{N_V} \times m}}}}^{V}$, respectively, Eq. (11) can be rewritten as

13 $$\begin{gathered} {\varvec{I}}^{\prime}(\theta )=\left[ {\begin{array}{*{20}{c}} {{\varvec{I}}^{\prime}(\theta )_{{{N_S} \times m}}^{S}} \\ {{\varvec{I}}^{\prime}(\theta )_{{{N_V} \times m}}^{V}} \end{array}} \right]=\left[ {\begin{array}{*{20}{c}} {\varvec{\alpha}\left( \theta \right)_{{{N_S} \times n}}^{S}{{(\varvec{\varPsi}_{{n \times n}}^{S})}^T}{{\mathbf{\Phi }}_{n \times m}}} \\ {\varvec{\alpha}\left( \theta \right)_{{{N_V} \times n}}^{V}{{(\varvec{\varPsi}_{{n \times n}}^{V})}^T}{{\mathbf{\Phi }}_{n \times m}}} \end{array}} \right] \hfill \\ \begin{array}{*{20}{c}} {}&{} \end{array}=\left[ {\begin{array}{*{20}{c}} {\varvec{\alpha}\left( \theta \right)_{{{N_S} \times n}}^{S}}&0 \\ 0&{\varvec{\alpha}\left( \theta \right)_{{{N_V} \times n}}^{V}} \end{array}} \right]\left[ {\begin{array}{*{20}{c}} {{{(\varvec{\varPsi}_{{n \times n}}^{S})}^T}}&0 \\ 0&{{{(\varvec{\varPsi}_{{n \times n}}^{V})}^T}} \end{array}} \right]\left[ {\begin{array}{*{20}{c}} {{{\mathbf{\Phi }}_{n \times m}}} \\ {{{\mathbf{\Phi }}_{n \times m}}} \end{array}} \right] \hfill \\ \end{gathered}$$

in which $\varvec{\varPsi}_{{n \times n}}^{S}$ and $\varvec{\varPsi}_{{n \times n}}^{V}$ are sparse matrices applied to conducting surface *S* and the dielectric regions *V*, respectively. In order to obtain excellent sparsity, the Hammett orthogonal basis^16^ and FFT basis^18^ are selected as $\varvec{\varPsi}_{{n \times n}}^{S}$ and $\varvec{\varPsi}_{{n \times n}}^{V}$, respectively, and unknown $\varvec{\alpha}\left( \theta \right)_{{{N_S} \times n}}^{S}$and $\varvec{\alpha}\left( \theta \right)_{{{N_V} \times n}}^{V}$ are reconstructed by the recovery algorithm in the next section.

### [Step 3] reconstructing the actual wide angle response

Since the sparsity of $$\varvec{\alpha}\left( \theta \right)_{{{N_t} \times n}}^{t}\begin{array}{*{20}{c}} {}&{(t=S,V)} \end{array}$$, the solution problem of underdetermined Eq. (13) can be converted into the *L*-norm optimization problem as

14 $${\mathbf{\hat {\alpha }}}{\text{(}}\theta {\text{)}}_{{{N_t} \times n}}^{t}{\text{=arg}}\hbox{min} ||\varvec{\alpha}\left( \theta \right)_{{{N_t} \times n}}^{t}|{|_L}\begin{array}{*{20}{c}} {}&{\left( {t=S,V} \right)} \end{array}$$

15 $$s.t.{\text{ }}\varvec{\alpha}\left( \theta \right)_{{{N_t} \times n}}^{t}{({\mathbf{\Psi }}_{{n \times n}}^{t})^{\text{T}}}{{\mathbf{\Phi }}_{n \times m}}={\varvec{I}}^{\prime}\left( \theta \right)_{{{N_t} \times m}}^{t}\begin{array}{*{20}{c}} {}&{\left( {t=S,V} \right)} \end{array}$$

For the execution of equations (14), (15), Orthogonal Matching Pursuit (OMP)^21^ is used as a recovery algorithm to reconstruct $$\varvec{\alpha}\left( \theta \right)_{{{N_t} \times n}}^{t}\begin{array}{*{20}{c}} {}&{\left( {t=S,V} \right)} \end{array}$$,and the actual response $${\varvec{I}}\left( \theta \right)_{{{N_t} \times m}}^{t}\begin{array}{*{20}{c}} {}&{\left( {t=S,V} \right)} \end{array}$$ can be obtained by solving Eq. (12).

## Computational complexity analysis

As we know, the classical VSIE method can easily analyze the scattering characteristics of composite conductive media objects with arbitrarily complex geometry. In other words, the VSIE method can handle arbitrary unknown ratios between dielectric region and conductor surface through the application of meshing techniques. However, the computational complexity formulation inherently varies depending on the specific ratios of unknowns allocated to dielectric region versus conductor surface, which accounts for the differences in algorithmic efficiency observed under different configuration scenarios. This distinction is principally evidenced in two aspects, as follows.

On the one hand, for composite structures where dielectric region unknowns significantly outnumber conductor surface unknowns, the VSIE solver with precorrected-FFT (p-FFT) acceleration demonstrates computational complexity comparable to VIE implementations, both of which are ${\rm O}(sN{\log _2}(N))$^12^ where *s* represents the iteration count. For wide-angle electromagnetic scattering problem, the complexity of solving VSIE repeatedly for *n* times by the traditional method is ${\rm O}(nsN{\log _2}(N))$. In the proposed method, the computational complexity consists of two parts. One part is solving VSIE for *m* (*m* < *n*) times to obtain *m* measurements, and its computational complexity is${\rm O}(msN{\log _2}(N))$. The other part comes from solving L-norm optimization problem by OMP, whose computational complexity is ${\rm O}(pmnN)$, where *p* is the sparse coefficient. Compared with the traditional method, the reduction rate of computational complexity from the proposed method can be estimated as

16 $$\eta =\frac{{smN{{\log }_2}(N)+pmnN}}{{snN{{\log }_2}(N)}}=\frac{m}{n}+\frac{{pm}}{{s{{\log }_2}\left( N \right)}}$$

Generally, *m* < < *n*, *pm* < < *s*.

On the other hand, different from the above composite targets, for composite objects with comparable unknowns between the dielectric region and the conductive surface, such as thin-coated metal targets, the computational complexity of solving VSIE using the pre-corrected FFT is similar to that of solving SIE, which is ${\rm O}(s{N^{1.5}}{\log _2}(N))$^12^ where *s* is the number of iterations. Furthermore, in terms of the efficient processing of wide-angle electromagnetic scattering by the proposed method, the computational complexity analysis for the targets of this section follows the same framework as that of the composite targets discussed earlier.This similarity enables the estimation of the computational complexity reduction rate as

17 $$\eta =\frac{{sm{N^{1.5}}{{\log }_2}(N)+pmnN}}{{sn{N^{1.5}}{{\log }_2}(N)}}=\frac{m}{n}+\frac{{pm}}{{s{N^{0.5}}{{\log }_2}\left( N \right)}}$$

In a word, although the computational complexity of the proposed method increases the part of the recovery algorithm compared with the traditional method, the computational cost of the recovery algorithm is less than that of the repeated computation saved by the proposed method, so the efficiency of the proposed method can still be guaranteed.

## Numerical results

In this section, the traditional VSIE and the proposed VSIE-CS are used to calculate the monostatic RCS of various composite conducting-dielectric objects, respectively. To reflect the advanced nature of the proposed method, both the VSIE-CS and VSIE methods are accelerated with the help of the fast algorithm P-FFT method. For all examples in this article, incident angles are uniformly discretized from 0° to 180° with a 1° angular step. Considering the balance between the recovery accuracy and speed of CS algorithm^16,18^ the proposed VSIE-CS selects the number of repeated calculations *m* = 30 to compare with *n* = 181 in traditional VSIE method. All calculation examples are performed on an Intel core i7 personal computer (CPU@2.4 GHz, RAM:16 GHz).

**Fig. 1:**
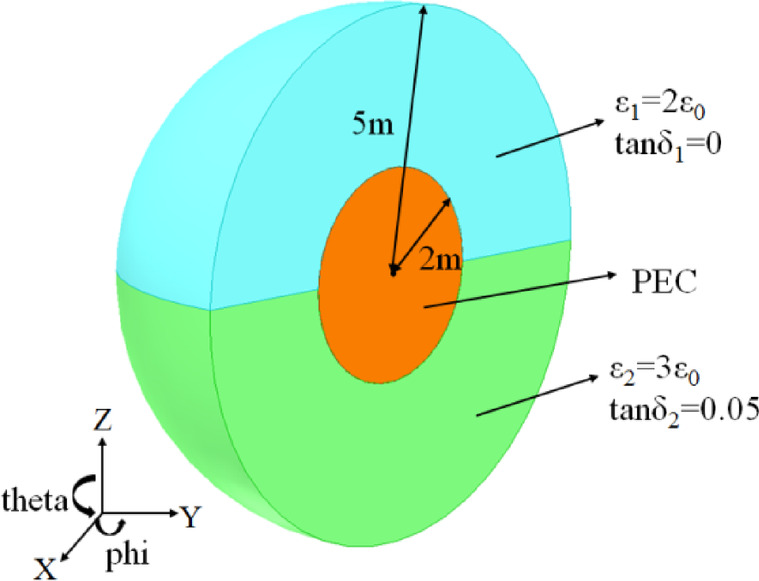
Geometry of Composite Conducting-Dielectric sphere.

**Fig. 2:**
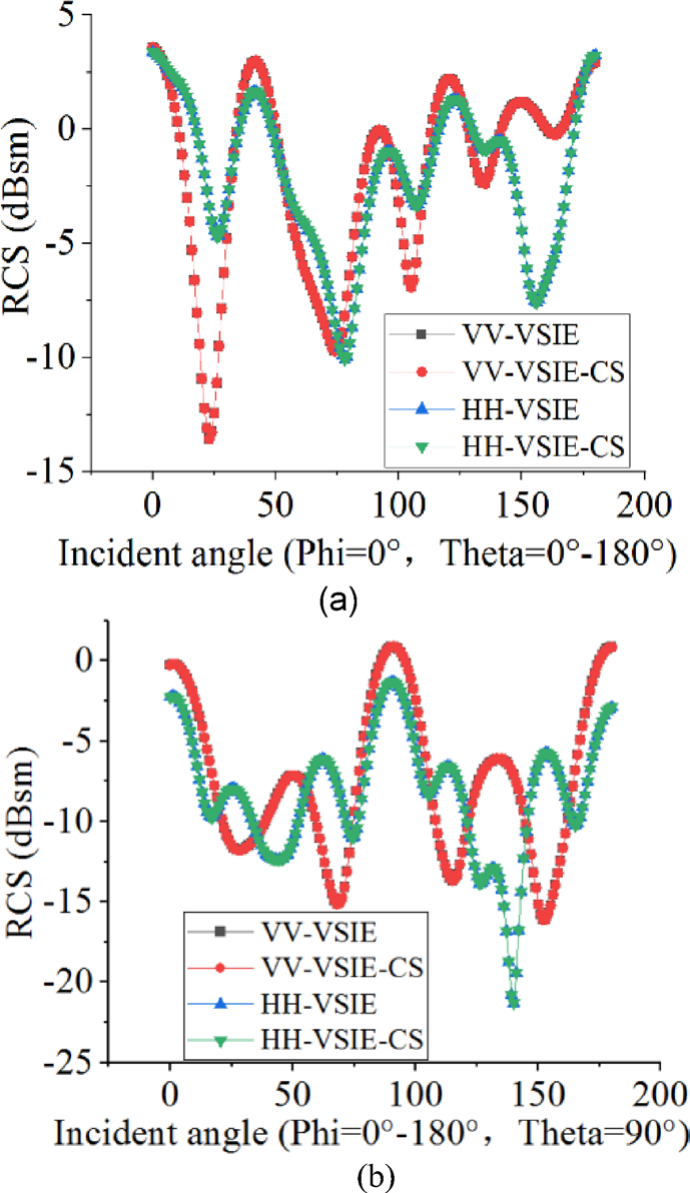
Monostatic RCS of the Composite Conducting-Dielectric sphere.

For the first example, we consider a composite conducting-dielectric sphere, which constructed by a PEC sphere with a radius of 2 m nested within two different material hemispheres with a radius of 5 m, where permittivity ε_1_ = 2ε_0_, loss tangent tanδ_1_ = 0, permittivity ε_2_ = 3ε_0_, loss tangent tanδ_2_ = 0.05, respectively, as shown in Fig. 1. Vertically polarized (VV) and horizontally polarized (HH) plane waves with a frequency of 300 MHz are used to irradiate the composite sphere, respectively. The dielectric region of the sphere is discretized into 19,381 tetrahedrons and the conducting surface into 502 triangular patches, yielding a total number of 40,762 unknowns. The monostatic RCS of the phi = 0° plane and theta = 90° plane from the composite sphere are shown in Fig. 2(a) and (b), respectively, and the numerical results calculated by VSIE and the VSIE-CS have good agreement at VV and HH polarizations. As can be seen from Table 1, compared with VSIE, the VSIE-CS method significantly reduces CPU time, while the peak memory consumption of the two methods keep the same level.

**Table 1:** The calculated data for the sphere.

Incident angle	Polarization	Method	Volume Unknowns	Surface Unknowns	The number of repeated calculations	CPU Time(s)	Peak memory (MB)
phi = 0°, theta-0°−180°	VV	VSIE	40,009	753	*n* = 181	887.7	1693.2
		VSIE-CS			*m* = 30	379.8	1627.4
	HH	VSIE	40,009	753	*n* = 181	821.8	1682.6
		VSIE-CS			*m* = 30	352.7	1654.8
phi = 0°−180°, theta = 90°	VV	VSIE	40,009	753	*n* = 181	879.1	2730.2
		VSIE-CS			*m* = 30	391.9	2763.6
	HH	VSIE	40,009	753	*n* = 181	895.2	2724.7
		VSIE-CS			*m* = 30	389.9	2742.9

**Fig. 3:**
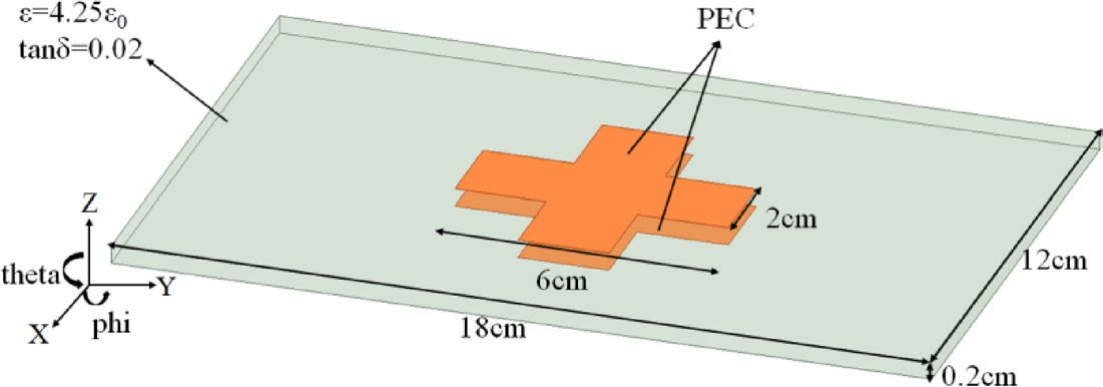
Geometry of FSS based on FR-4 dielectric substrate.

**Fig. 4:**
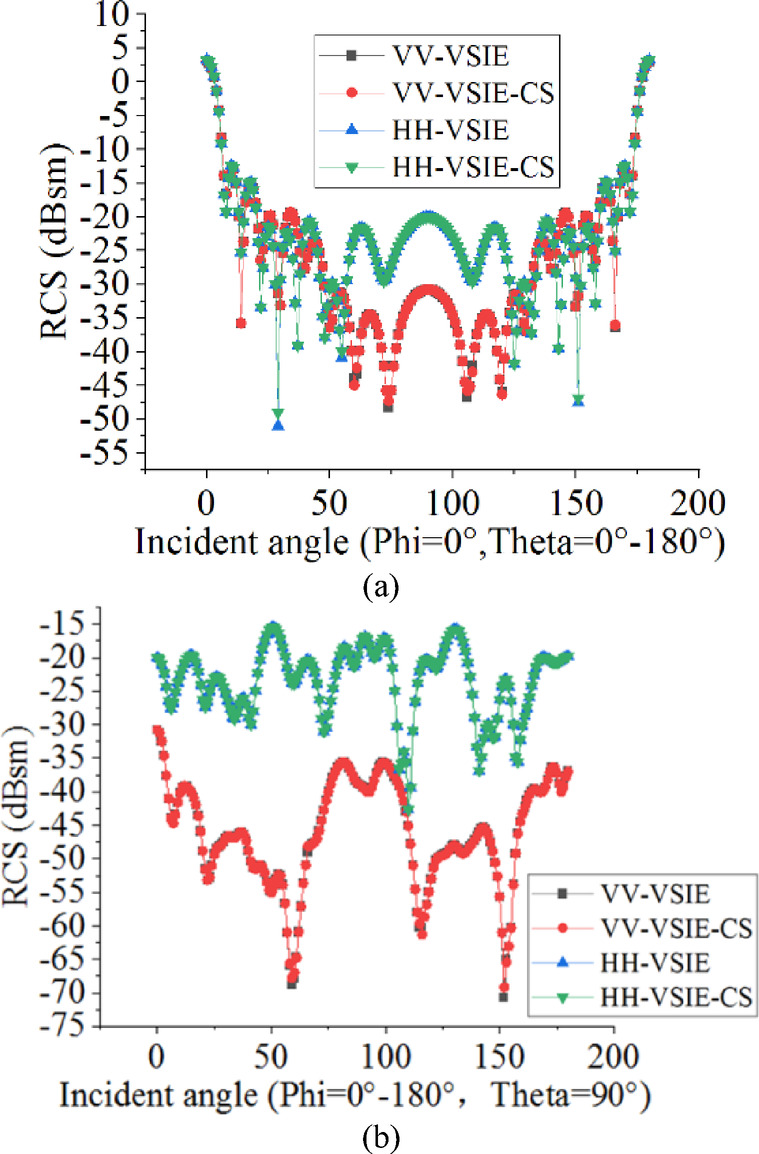
Monostatic RCS of the FSS based on FR-4 dielectric substrate.

In the second example, considering a Frequency Selective Surface (FSS) based on FR-4 dielectric substrate (ε = 4.25ε_0_, tanδ = 0.02) is illuminated by a plane waves at 10 GHz from VV and HH polarizations, respectively. The FSS consists of an FR4 dielectric substrate (18 cm*12 cm*0.2 cm) and two cross-shaped PEC patches printed on the upper and lower sides of the substrate, and its geometric structure and specific dimensions are shown in Fig. 3. In this example, the region of the dielectric substrate and the surface of the PEC patches are discretized into 11,078 tetrahedrons and 1094 triangles, respectively, resulting in a total of 27,339 unknowns.Figure 4(a) and (b) show the monostatic RCS of the phi = 0° plane and theta = 90° plane from the FSS, respectively. It can be seen from the Figure 4 that the results achieved by the proposed VSIE-CS agree very well with the traditional method VSIE, under different polarization conditions. The calculated data are shown in Table 2, in which the CPU time of VSIE-CS decreases by about 55% compared with VSIE under the same unknowns.

**Table 2:** The calculated data for the FSS.

Incident angle	Polarization	Method	Volume Unknowns	Surface Unknowns	The number of repeated calculations	CPU Time(s)	Peak memory (MB)
phi = 0°, theta-0°−180°	VV	VSIE	25,698	1641	*n* = 181	825.9	961.4
		VSIE-CS			*m* = 30	369.8	967.6
	HH	VSIE	25,698	1641	*n* = 181	825.7	961.4
		VSIE-CS			*m* = 30	369.1	966.8
phi = 0°−180°, theta = 90°	VV	VSIE	25,698	1641	*n* = 181	848.8	961.4
		VSIE-CS			*m* = 30	373.3	965.4
	HH	VSIE	25,698	1641	*n* = 181	816.9	961.4
		VSIE-CS			*m* = 30	361.9	965.6

**Fig. 5:**
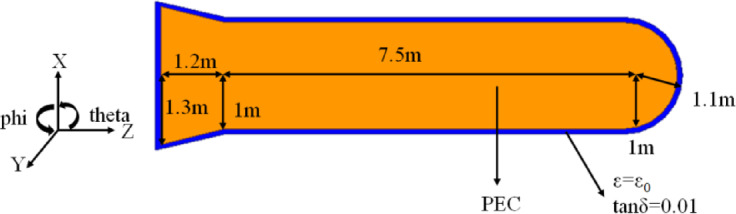
Geometry of Composite Conducting-Dielectric Missile.

**Fig. 6:**
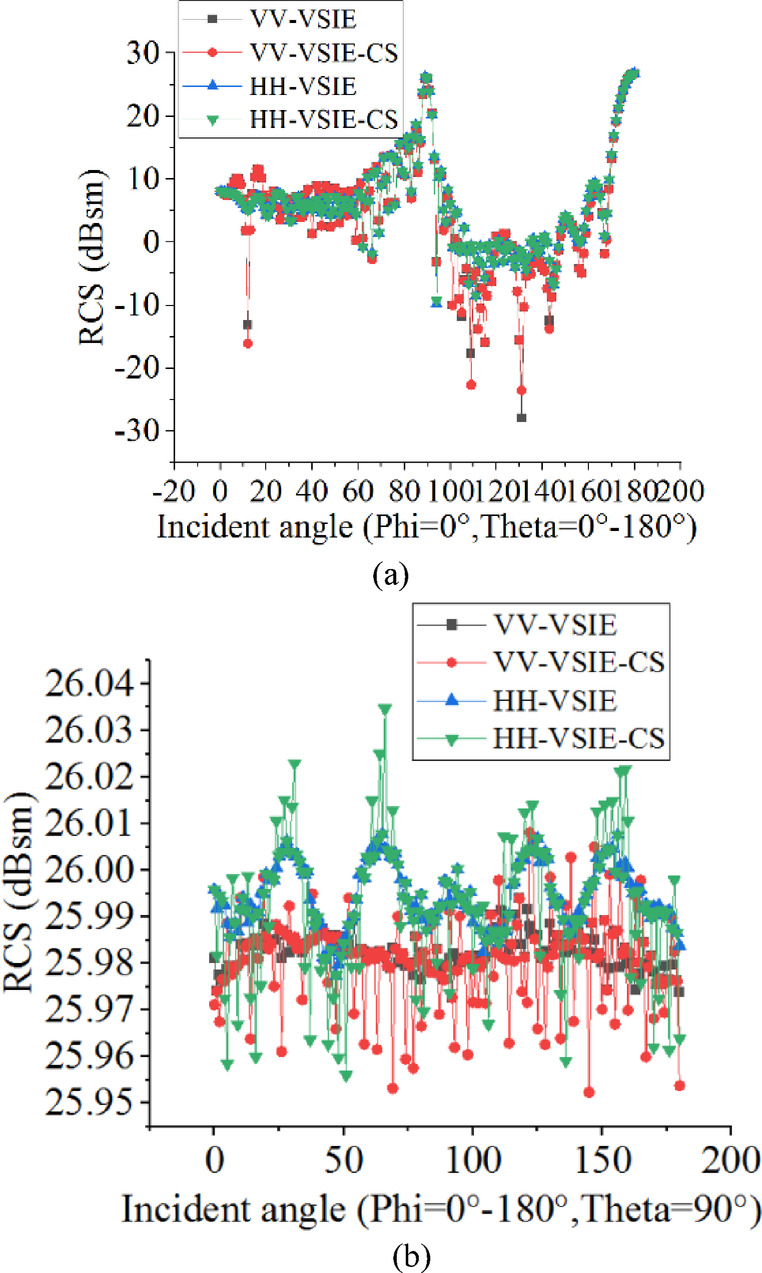
Monostatic RCS of the Composite Conducting-Dielectric Missile.

In the final example, the EM scattering from a Composite Conducting-Dielectric Missile is considered. As shown in Fig. 5, the composite target is constructed from a PEC missile body covered with dielectric material (ε = ε_0_, tanδ = 0.01). Its total length is 9.9 m, and the inner and outer radius of the composite warhead is 1 m and 1.1 m, respectively. The geometric structure and specific dimensions of the scatterer are described in the Figure 5. The work frequency of incident plane wave is 300 M Hz, and the total number of unknowns is 79,923, the dielectric shell is modeled by 25,266 tetrahedrons, and the PEC surface is modeled by17584 triangles. In Fig. 6, the results of monostatic RCS calculated by the VSIE-CS and VIE methods are compared. For VV and HH polarizations, the two methods have a good agreements in phi = 0° plane, as shown in Figure 6(a). In Figure 6(b), under the above same conditions, the maximum error of the results from the two methods in theta = 90° plane is only 0.05dB, which can also explain the accuracy of the proposed method. In Table 3, the calculated data validated advantage of the VSIE-CS method for electrical large object.

**Table 3:** The calculated data for the Missile.

Incident angle	Polarization	Method	Volume Unknowns	Surface Unknowns	The number of repeated calculations	CPU Time(s)	Peak memory (MB)
phi = 0°, theta-0°−180°	VV	VSIE	53,547	26,376	*n* = 181	7601.1	777.6
		VSIE-CS			*m* = 30	2318.9	778.9
	HH	VSIE	53,547	26,376	*n* = 181	5541.4	783.1
		VSIE-CS			*m* = 30	1708.7	785.9
phi = 0°−180°, theta = 90°	VV	VSIE	53,547	26,376	*n* = 181	3902.3	788.8
		VSIE-CS			*m* = 30	1218.2	783.5
	HH	VSIE	53,547	26,376	*n* = 181	3653.7	776.4
		VSIE-CS			*m* = 30	1153.2	779.6

## Conclusion

In this article, the proposed VSIE-CS method is applied to improve the analysis efficiency of wide-angle electromagnetic scattering from Composite Conducting- Dielectric Objects. The numerical results verify the proposed method has advantages of accelerating calculation by reducing the number of repeated calculations of matrix equations on the premise of ensuring the accuracy.

## Data Availability

All data required to evaluate the findings of this work is available in the presented paper. Additional data related to this work may be requested from the corresponding author.
